# Adult Outcomes of Childhood Wheezing Phenotypes Are Associated with Early-Life Factors

**DOI:** 10.3390/jpm14121171

**Published:** 2024-12-22

**Authors:** Sophie Carra, Hongmei Zhang, Luciana Kase Tanno, Syed Hasan Arshad, Ramesh J. Kurukulaaratchy

**Affiliations:** 1 Respiratory Biomedical Research Centre, University Hospital Southampton, Southampton SO16 6YD, UKsha@soton.ac.uk (S.H.A.); 2 The David Hide Asthma and Allergy Research Centre, St Mary’s Hospital, Newport, Isle of Wight PO30 5TG, UK; 3 Division of Epidemiology, Biostatistics, and Environmental Health, School of Public Health, University of Memphis, Memphis, TN 38111, USA; hongmei.zhang@memphis.edu; 4 Desbrest Institute of Epidemiology and Public Health, UMR UA11 University of Montpellier—INSERM, 34295 Montpellier, France; l-kasetanno@chu-montpellier.fr; 5 Division of Allergy, Department of Respiratory Medicine & Allergy, University Hospital of Montpellier, 34295 Montpellier, France; 6 WHO Collaborating Centre on Scientific Classification Support, 34295 Montpellier, France; 7 Clinical and Experimental Sciences, Faculty of Medicine, University of Southampton, Tremona Road, 810, F-Level, Southampton SO16 6YD, UK

**Keywords:** asthma, bronchial hyperresponsiveness, lung function, phenotypes, wheezing

## Abstract

**Introduction:** While the phenotypic diversity of childhood wheezing is well described, the subsequent life course of such phenotypes and their adult outcomes remain poorly understood. We hypothesized that different childhood wheezing phenotypes have varying longitudinal outcomes at age 26. We sought to identify factors associated with wheezing persistence, clinical remission, and new onset in adulthood. Methods: Participants were seen at birth and at 1, 2, 4, 10, 18, and 26 years in the Isle of Wight Birth Cohort (*n* = 1456). Information was collected prospectively on wheeze prevalence and phenotypic characteristics at each assessment. Wheeze phenotypes at 10 years were defined as participants wheezing (CW10) or not wheezing at 10 (CNW10). Multivariable regression analyses were undertaken to identify factors associated with wheezing persistence/remission in CW10 and wheeze development in CNW10 at age 26 years. **Results:** Childhood wheezing phenotypes showed different subsequent outcomes and associated risk factors. Adult wheeze developed in 17.8% of CNW10. Factors independently associated with adult wheeze development in CNW10 included eczema at age 4 years, family history of rhinitis, and parental smoking at birth. Conversely, 56.1% of CW10 had remission of wheeze by 26 years. Factors predicting adult wheezing remission in CW10 included absence of both atopy at age 4 years and family history of rhinitis. **Conclusion:** Early-life factors influence adult outcomes for childhood wheezing phenotypes, both with respect to later development of adult wheezing in asymptomatic participants and of wheeze remission in childhood wheezers. This suggests potential areas that could be targeted by early-life interventions to alleviate adult disease burden.

## 1. Introduction

Wheezing is commonly encountered in childhood, reflecting both virus-associated and multitrigger effects. While not synonymous with asthma in childhood, childhood wheezing, if persistent, may reflect the presence of childhood asthma [1]. Phenotypic classifications of childhood wheezing have provided key insights into the diverse nature of this symptom, its association with childhood health outcomes and its association with diagnosed asthma [2,3,4,5].

A landmark temporally defined phenotypic classification from the Tucson Children’s Respiratory Study (TCRS) [6], categorized participants into four distinct phenotype groups based on their wheezing status during the first six years of life. In the Isle of Wight Birth Cohort (IOWBC), we adapted this classification for timepoints over the first decade of life [7]. While an enhanced understanding of childhood wheeze and asthma has evolved from such phenotypic approaches [2,3,4,5], a key gap in knowledge is how childhood wheezing phenotypes track across the wider life course and to what extent they are related to adult wheezing and asthma status. A small number of studies have previously assessed the trajectory of childhood wheeze and asthma into adolescence and adulthood [8,9,10,11,12,13,14,15,16,17,18,19]. Early-life factors recognized to be associated with persistence of early-life wheezing into adolescence include family history of asthma or atopy, personal history of atopic conditions, early-life allergic sensitization, demonstration of Type-2 (T2) inflammatory signals, and the frequency and severity of wheezing [8]. Studies of disease trajectories into adulthood have identified a variety of patterns ranging from remission to persistence and incident disease. They have shown that most children with mild early-life wheezing outgrow their disease by adulthood [5,19,20]. Conversely, emerging evidence indicates that more severe childhood asthma is often associated with more severe adulthood asthma [20,21,22]. Studies of wheeze/asthma pathways from childhood to adulthood have identified persistent trajectories [17,18,19,20]. These have shown association with more severe asthma, impaired lung function, bronchial hyperresponsiveness and allergic sensitization in childhood, and adult T2 expression and lung function impairment [17,18,19,20]. However, none of these studies have examined childhood wheezing phenotypes within the framework of longitudinal birth cohorts. This knowledge gap is highly relevant given the increasingly recognized concepts of the early-life origins of adult wheeze/asthma and the tracking of impaired lung function from childhood into adulthood in some childhood asthmatics [5,8,16,19].

In this paper, we determine the longitudinal outcomes at the age of 26 years for childhood wheezing phenotypes defined at 10 years of age in the IOWBC [7,23,24,25,26]. Our hypotheses were that (a) childhood wheezing phenotypes show differing associations with young adult wheeze, (b) a proportion of childhood wheezers show remission by adulthood, (c) a proportion of participants who did not wheeze start to do so by young adulthood, and (d) factors in early life and adolescence are associated with wheeze remission, persistence, and new onset by adulthood.

## 2. Methods

### 2.1. The Study Cohort

**Figure 1:**
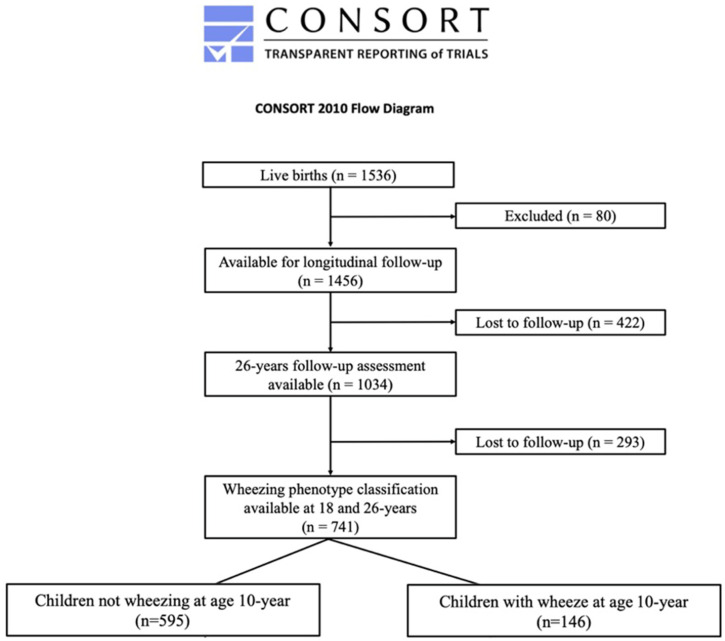
CONSORT diagram showing participants recruited to the study and how their wheezing phenotypes were classified.

In 1989, a whole-population-based birth cohort was initiated on the Isle of Wight to investigate the natural history of asthma and allergic conditions across the life course [7]. The study was conducted in accordance with the Declaration of Helsinki and approved by the Institutional Review Board (or Ethics Committee) of Isle of Wight Post Graduate Medical Federation (No 05/89; dated 22 August 1988).which was updated at each subsequent follow-up. Consent was secured from 1456 out of 1536 participants born between January 1989 and February 1990. The cohort was followed from birth through follow-ups at ages 1, 2, 4, 10, 18, and 26 years (Figure 1) [7,23,24,25,26]. Detailed parent-completed questionnaires assessed asthma and allergies in early childhood. From age 10 years, International Study of Asthma and Allergies in Childhood (ISAAC) [27] questionnaires evaluated symptoms, alongside detailed supplementary questionnaires at each study visit. A cut-off of 18 years or greater was used to define adult status.

### 2.2. Variables

Current wheeze was defined as “wheeze or whistling in the last 12 months” as reported by either the parent (in childhood) or the participant (in adolescence/adulthood) in response to investigator-administered ISAAC questionnaires. Asthma was diagnosed as a composite diagnosis of “physician-diagnosed asthma ever plus either current wheeze in the past 12 months or taking asthma medications in the past 12 months”.

Spirometry was measured at ages 10, 18, and 26 years. Absolute values for forced expiratory volume in 1 s (FEV_1_), forced vital capacity (FVC), and forced midexpiratory flow (FEF_25–75_) were used for analysis. Bronchial hyperresponsiveness (BHR) was assessed from age 10 onwards using methacholine challenge in a subgroup. The methodology for spirometry and bronchial challenge has been previously described [7,28]. FeNO (fractional exhaled nitric oxide) was measured at 18 and 26 years.

Skin prick testing (SPT) from age 4 assessed atopy (with mean wheal diameter >3 mm greater than the negative control to individual allergen defining sensitization) using a standardized battery of common allergens (ALK, Hørsholm, Denmark). Serum total IgE (Immunoglobulin E) was measured at 10 and 18 years.

BHR was categorized aligning with the American Thoracic Society BHR guideline categories, with “Definite BHR” classified as provoking methacholine concentration causing 20% fall in FEV_1_ (PC_20_) < 4 mg/mL [28]. We also used a continuous dose–response slope (DRS) estimated by log-transformed least-square regression of FEV_1_ changes to methacholine doses, since not all subjects demonstrated a 20% fall in FEV_1_ during the bronchial challenge. A transformation of Log10 (DRS+ 10) was required to satisfy the distributional assumption of normal data.

### 2.3. Statistical Analysis

Data were entered into SPSS (Statistical Package for the Social Sciences) (v24, IBM statistics, Armonk, USA) using a double-entry method and analysed by the R software (R Core Team, Vienna, Austria, 2022).

First, we undertook descriptive assessment of baseline characteristics and outcomes at 10 years for both CNW10 (participants not wheezing at age 10 years) and CW10 (participants wheezing at age 10 years). We then performed similar analyses for those participants (CNW10 and CW10) again at 26 years of age. For these analyses, categorical variables were assessed by chi-square tests (with Fisher’s exact test where low cell counts occurred), while continuous variables were assessed by appropriate parametric tests (e.g., *t*-tests) when the normality assumption reasonably held or nonparametric (e.g., Mann–Whitney U for two-sample comparisons) when the assumption was violated.

Next, we implemented time-lagged univariate analyses to assess the association of each of the early-life factors with wheeze status at 26 years for CNW10 and CW10 for the purpose of selecting potentially informative variables for subsequent analyses. These early-life factors included family history of asthma, eczema, rhinitis, low birthweight <2.5 kgs, exclusively breastfed newborn for at least 3 months, lower socioeconomic status at birth, recurrent chest infections at 1 and 2 years of age, parental smoking at birth and at 4 years of age, eczema at 4 years of age, atopy at 4 years of age, and rhinoconjunctivitis at 4 years of age. In this analysis, factors with *p*-value < 0.2 were then selected and included in multivariable time-lagged regression models [29] to examine their associations with (a) wheezing persistence in CW10 to age 26 years and (b) wheezing development at age 26 years in CNW10.

In the final phase, we used backward variable selection to identify early-life factors associated with the two outcomes, wheezing persistence and wheezing development. Given the use of longitudinal data and the multivariable regression modelling with prospective time-order, we minimized potential reverse causation.

## 3. Results

### 3.1. Study Population Description

A total of 1034 IOWBC participants (70.9% of the original birth cohort) were assessed at 26 years. Of these, 741 (71.7%) were reassessed at all 3 (10-, 18-, and 26-year) follow-ups from 10 years onwards. That subgroup formed the study population for this analysis (Figure 1). Subjects included in this analysis differed from the 293 (28.3%) excluded because of missing follow-up participation only with respect to male sex (*p* < 0.001; Table 1).

**Figure 2:**
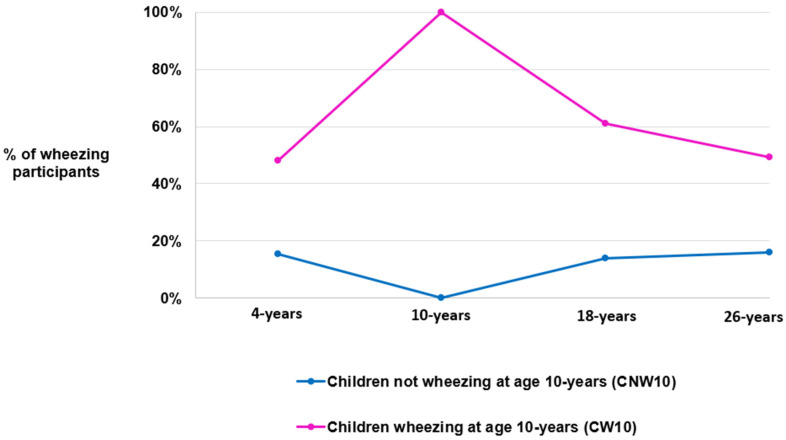
Changes in the percentage of individuals with an active current wheezing status over time, categorized by their childhood wheeze phenotype group.

Most of this study population was in the CNW10 group (*n* = 595, 80.3%), while 19.7% (*n* = 146) were in the CW10 group (Figure 2). Among CNW10, wheeze prevalence rose from 0% to about 20% by age 26 years. Conversely, among CW10, wheeze prevalence fell from 100% to about 50% at 26 years (Figure 2).

### 3.2. Comparison of Characteristics for CW10 and CNW10 in Childhood and Adulthood

Characteristics and outcomes with statistical significance between CW10 and CNW10 at 10 and 26 years are shown in Table 1. Statistically insignificant findings are provided in Table 2. CW10 was associated with higher current wheeze at 26 years and higher asthma prevalence at 10 and 26 years (*p* < 0.001, Table 1). At the age of 18 years, the prevalence of atopy was higher than at the age of 10 years in the two groups, CNW10 and CW10. (Table 1). Similar findings were found for current rhinitis. Current eczema was also higher at 10 years in CW10 than CNW10 (*p* = 0.046), but this difference had disappeared by 26 years of age. CW10 had higher prevalence of asthma treatment than CNW10 at 10 years (*p*< 0.001) and at 26 years (*p* = 0.051) (Table 1). Treatment with inhaled corticosteroids was greater in the CW10 group at 10 (*p* = 0.0013) but not at 26 years (*p* = 0.0802) (Table 1). Current active and passive smoking did not differ between CW10 and CNW10 at 10 years of age (Table 2).

### 3.3. Lung Function

#### FEV_1_-FVC-FEV_1_/FVC

**Figure 3:**
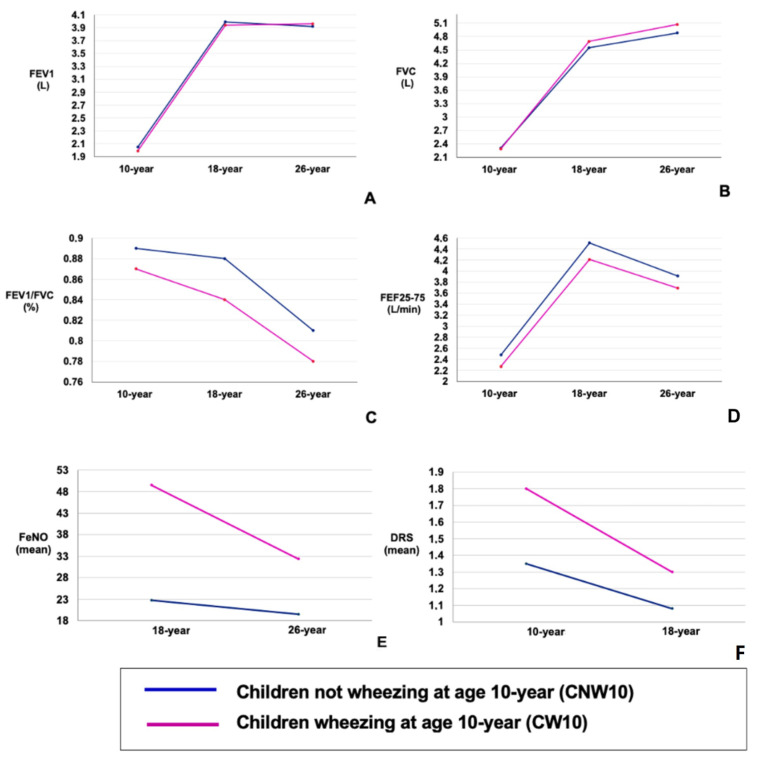
(**A**–**F**): Longitudinal trajectories in prebronchodilator lung function at 10, 18, and 26 years of age for childhood wheezing phenotypes. Data are presented for childhood wheezing phenotypes. (**A**) Longitudinal prebronchodilator FEV1 (L) outcomes. (**B**) Longitudinal prebronchodilator FVC (L) outcomes. (**C**) Longitudinal prebronchodilator FEV1/FVC (%) outcomes. (**D**) Longitudinal prebronchodilator FEF25–75 (L/min) outcomes. (**E**) Longitudinal mean FeNO outcomes. (**F**) Longitudinal mean of dose–response scale at methacholine challenge outcomes.

For both groups, from 10 to 26 years, FEV_1_ showed a pattern of an adolescent increase followed by a plateau, while FVC had a pattern of consistent increase, FEF_25–75_ demonstrated an adolescent increase followed by decline in young adulthood, and FEV_1_/FVC seemed to decrease (Figure 3). Compared with CW10, CNW10 showed higher FEV_1_ at 10 years (*p* = 0.04), FEV_1_/FVC at 10 (*p* = 0.0002), 18 (*p* < 0.0001), and 26 years (*p* < 0.0005), and FEF_25–75_ at 10 (*p* = 0.0002) and 18 years (*p* = 0.01) (Table 2). However, at 26 years, FVC was higher in the CW10 group (*p* = 0.04) (Table 2). The FEV_1_, FVC, and FEV_1_/FVC ratio changed between ages 10–18 and 18–26 for the CW10 and CNW10 groups, as shown in Table 3.

### 3.4. FeNO

At each age, FeNO was higher in those from the CW10 group than in those from the CNW10 group (Table 2). Both the CW10 and CNW10 groups showed declining trends between 18 and 26 years for FeNO. This decrease differed between the two groups, being more pronounced for the 26–18-year part in the CW10 group than in the CNW10 group (*p* < 0.002) (Table 3).

### 3.5. Bronchial Hyperresponsiveness

The proportion of participants with BHR (PC_20_ <4 mg/mL) was greater in the CW10 group at 10 years compared with the CNW10 group (*p* < 0.001) (Table 1). DRS was greater (indicating higher BHR) in CW10 at both ages (Table 2). A declining trend for DRS seemed to exist for both CW10 and CNW10 from 10 to 18 years. Figure 3. The decline in the DRS rate was more pronounced in the CW10 group between 18 and 10 years of age than in the CNW10 group (*p* < 0.002) (Table 3).

### 3.6. Longitudinal Wheeze Outcomes of CW10 and CNW10 at Age 26 Years and Associated Factors

For further longitudinal outcome analysis, 9.2% (55/595) of participants in the CNW10 group and 9.6% of participants (14/146) in the CW10 group were excluded from assessment because of transitory isolated wheezing in those participants at 18 years (see Table 4 notes).

Adult wheeze developed in 17.8% (96/540) of CNW10. Among CNW10, compared with those who wheezed at 26 years, those who did not wheeze at 26 years had lower prevalence of asthma and rhinitis at 26 years (Table 4). In the CW10 group, 43.9% (58/132) continued wheezing into adulthood, while 56.1% (74/132) experienced symptom remission. Compared with participants who lost wheezing by adulthood, CW10 subjects who continued wheezing up to adulthood had higher prevalences of wheezing, asthma diagnosis, rhinitis, atopy, asthma treatment at 10 and 26 years, and BHR at 10 years, as well as higher BMI (body mass index) at 26 years (Table 4). Prevalence of eczema was higher at 26 years among those still wheezing at 26 years compared with those with wheeze remission (Table 4). Other assessed variables that did not show statistically significant differences between the studied groups are presented in Table 3 and Table 4.

### 3.7. Early-Childhood Risk Factors for Wheezing Appearance/Reappearance or Remission in Adulthood

When considering associations with early-life risk factors, CNW10 with wheezing in adulthood had higher prevalences of respiratory infections at 2 years, eczema at 4 years, and family history of rhinitis (Table 4). Conversely, among CW10, those who lost wheezing by adulthood had lower prevalences of atopy at 4 years and positive family history of rhinitis compared with the other group of participants who continued to wheeze (Table 4).

The factors that met the inclusion criterion (*p*-value < 0.2) in the multivariate model for adult wheezing development were recurrent chest infections by age 2, eczema by age 4, family history of rhinitis, parental tobacco smoking at birth, and rhinitis at age 4. For wheezing remission in adulthood, the variables that qualified for inclusion were atopy at age 4, family history of rhinitis, low birth weight (<2.5 kg), and exposure to tobacco smoke at birth. Following multivariable logistic regression analysis, factors adjusted for other covariates (Table 5A, notes) with the development of adult wheezing in CNW10 included a history of eczema at age 4 years (*p*-value < 0.0005), family history of rhinitis (*p*-value = 0.02), and parental tobacco usage at birth (*p*-value = 0.04) (Table 5A). Factors adjusted for other covariates (Table 5B, notes) with wheezing remission by adulthood in CW10 included a lack of atopy at age 4 years (*p* = 0.04) and the absence of familial predisposition to rhinitis (*p*-value = 0.0006) (Table 5B).

## 4. Discussion

To address our original hypotheses, we characterized wheezing phenotypes among 10-year-olds in the IOWBC, defined their longitudinal adult wheezing outcomes at 26 years, and identified associated risk factors for those outcomes. Our childhood wheezing phenotypes were defined by the presence or absence of current wheezing at 10 years, CW10 or CNW10. CW10 encompassed previously characterized late-onset and persistent childhood wheeze, while CNW10 represented transient and never-wheezed “early childhood phenotypes” [24]. With respect to our first hypothesis, we found that CW10 had higher prevalences of young adulthood wheeze, diagnosed asthma, worse airflow limitation, FeNO, and BHR than CNW10. Addressing our second hypothesis, half of CW10 no longer wheezed at 26 years. In the context of our fourth hypothesis, we found that such wheeze remission by age 26 years in CW10 was independently associated with absence of atopy at 4 years and lack of rhinitis family history. Conversely, we found that a majority (57%) of current wheezers at 26 years were asymptomatic at 10 years. Further, addressing our third hypothesis, we found that nearly one-fifth of CNW10 developed wheezing at 26 years. Returning to our fourth hypothesis, this outcome was independently associated with parental smoking at birth, eczema at 4 years, and rhinitis family history. While our findings showed clear associations with potentially relevant risk factors, they cannot be used to infer definitive causality. Nevertheless, associations of early-life factors with adult outcomes of childhood wheeze phenotypes provide opportunities to potentially intervene in early life to influence their subsequent life course impacts.

While wheeze and asthma are not synonymous entities, a diagnostic label of adult asthma infers the presence of a more significant wheezing state. Despite numerous studies suggesting that adult wheeze and asthma often originate in early childhood [5,8,19,30,31,32,33,34,35,36], few have used prospectively collected data to specifically investigate early-life risk factors related to adult-onset wheeze or asthma in the context of childhood wheezing phenotypic classification. Factors such as shorter duration of breastfeeding or having ≥ 2 siblings have been associated with higher rates of adult-onset asthma [37,38], but information on time of onset of wheeze/asthma and early-life factors was collected retrospectively and thus prone to recall bias. We identified that family history of rhinitis, parental smoking at birth, and eczema at age 4 years were associated with young-adult wheeze in those who were asymptomatic at 10 years. Previous studies have linked family history of rhinitis to a quadruple rise in the likelihood of wheeze or asthma development in childhood [39,40]. We found that rhinitis family history conferred a twofold increased risk of adult wheeze in asymptomatic 10-year-olds. Family history of rhinitis may be considered as a proxy for inherited predisposition to atopy and could be used as a marker to identify infants at high risk of asthma for early intervention [41]. Such family history of rhinitis may indicate a genetic or environmental predisposition to airway hyperresponsiveness, potentially driving chronic inflammation and shared inflammatory pathways between rhinitis and the lower respiratory tract. Passive smoke exposures in early life represent a clear modifiable risk factor [42,43]. They constitute a pivotal risk factor for respiratory ailments, stunted lung growth, manifestation of wheezing symptoms in childhood, and chronic obstructive pulmonary disease (COPD) in adulthood [42,44,45,46,47].

Furthermore, recently reported findings demonstrated associations of exposure to maternal smoking in pregnancy with accelerated lung function decline in adulthood [48]. Such findings align with our observed associations of early-life passive smoke exposure with new-onset wheezing in adulthood [49,50]. Our results further emphasize the need to eliminate exposure to tobacco smoke in early childhood, as the consequences can be far reaching and may appear several decades after exposure.

Eczema in infancy is a recognized risk factor for wheeze or asthma development in childhood [51]. Recent findings have indicated that the proposed linear progression of the atopic march [52] fails to encompass the diversity of allergic phenotypes and their trajectories. Nevertheless, atopic dermatitis frequently serves as the initial manifestation of a progressive atopic syndrome, and our results show that it also indicates risk for development of pulmonary symptoms in adulthood. It may simply reflect atopic predisposition of these participants; however, could prevention of eczema reduce the risk of adult wheeze or asthma? The administration of probiotics during pregnancy seemed to reduce the likelihood of developing eczema in childhood by approximately 20% [53], but it was not associated with a reduction in the onset of asthma symptoms [53,54]. Several studies have also assessed restoration of the epithelial skin barrier and its effect on the onset of pulmonary asthma symptoms, but none has yet shown a significant effect [55]. Further studies are needed to identify preventive strategies to target the expression of atopic diseases in the skin and the respiratory tract at the same time. One such strategy might be to correct an evolving Type 2 inflammatory imbalance by using prophylactic allergen immunotherapy [56].

Of wheezing subjects at age 10, 56.1% no longer had pulmonary symptoms at age 26, defined as clinical remission. Past findings have indicated that remission of childhood wheezing is more common with milder childhood wheezing [20,21,22]. They have also shown that clinical remission is less frequent for those with a label of childhood asthma and much less so for severe childhood asthma. This probably reflects that a diagnosis of childhood asthma aligns with a more severe wheezing phenotype that is more likely to persist. Previous studies have revealed that clinical remission of wheezing symptoms in childhood or adolescence is favoured by lower initial BHR, significant improvement in small-airway function, male sex, milder disease (with less frequent and severe symptoms), and less atopic sensitization [56,57,58]. One possible focus for preventive measures to promote clinical wheeze remission among childhood wheezers is on mitigation against allergen sensitization. Early-life allergic sensitization, especially multiple sensitizations, poses heightened risk of impaired lung function and enhanced BHR by adolescence [59]. Future primary prevention strategies might target infancy, leveraging early allergen exposure to induce immune tolerance [56]. Indeed, our recent primary prevention study of allergen immunotherapy suggested that early administration of house dust mite sublingual immunotherapy in high-risk, nonsensitized participants could potentially reduce childhood wheeze and asthma incidence [56]. Recently, we also developed the ASPIRE (asthma predictive risk score) system [60], aiming to predict adult asthma status from early childhood. Our present study is in line with the ASPIRE score, implying the same risk factors for persistent wheezing in adulthood (atopy at 4 years and a family history of rhinitis). This underscores the potential of early intervention based on a few routinely collected factors. Better prediction of disease outcome can lead to better therapeutic adherence and, consequently, a more favourable evolution of symptoms.

A core strength of our study is the rich longitudinal dataset that enabled extensive characterization of childhood wheeze phenotypes and their longitudinal outcomes. High cohort retention and nondifferential loss to follow-up in core parameters permitted meaningful investigation of long-term outcomes. One limitation of our study is a lack of objective data such as lung function in the first 4 years of life, though this was available from 10 years onwards. As recently shown in the Vitamin D Antenatal Asthma Reduction Trial, earlier lung function measures in the pre-school phase may have offered unique perspectives on subsequent respiratory morbidity and lung function [61]. As shown in other studies, impaired lung function trajectories may establish early in childhood, and factors associated with that merit attention in future studies [22]. Another limitation is that our population was very homogeneous, and replication in other populations is indicated to assess consistency of findings in other geographic areas and ethnicities. However, at present, comparable study cohorts for replication to age 26 are limited. Finally, as with any epidemiological study, our findings cannot infer causality but should be viewed as indicative of associations.

## 5. Conclusions

Our IOWBC study provides valuable insights into childhood wheezing outcomes up to age 26. A substantial proportion of CW10 individuals no longer experienced wheezing by age 26, while a significant number of CNW10 individuals developed wheezing during this period. Absence of pulmonary symptoms in late childhood does not guarantee their absence in adulthood. We have identified clinical factors in early childhood associated with the onset or clinical remission of wheezing in adults in a birth cohort. This underscores the importance of pursuing early-life interventions that could have profound and lasting impacts on life course wheezing outcomes.

## Figures and Tables

**Table 1:** Characteristics at 10 and 26 years with statistically significant differences between childhood wheeze phenotypes.

Variable If Categorical No (%) If Numeric Mean (Min–Max)	Children Not Wheezing at Age 10 Years (CNW10) (*n* = 595)	Children Wheezing at Age 10 Years (CW10) (*n* = 146)	*p*-Value
10 years			
Current asthma at 10 years	2.0% (12/594)	28.1% (38/135)	<0.00001 *
Current rhinitis at 10 years	12.2% (72/588)	39.7% (58/146)	<0.00001 *
Current eczema at 10 years	18.6% (110/590)	26.1% (38/146)	0.046 *
Atopic status at 10 years	20.1% (102/507)	58.7% (81/138)	<0.00001 *
Asthma treatment at 10 years	2.4% (13/532)	68.3% (99/145)	<0.00001 *
Bronchial hyperreactivity (BHR) at 10 years defined by PC20 <4 mg/ml (%)	12.1% (42/349)	48.9% (64/131)	<0.00001 *
26 years			
Current asthma at 26 years	7.2% (43/594)	46.9% (88/145)	<0.00001 *
Current wheeze at 26 years	16.1% (96/595)	49.3% (72/146)	<0.00001 *
Current rhinitis at 26 years	39.8% (237/595)	59.6% (87/146)	0.000016 *
Atopic status at 26 years	40.7% (144/354)	69.1% (65/94)	<0.00001 *

A chi 2 test was performed for each characteristic. Test made by Fisher’s exact test when necessary. * *p*-value for significance is 0.05.

**Table 2:** Prebronchodilator lung function, FeNO, and BHR dose–response slope characteristics of 10 years childhood wheezing phenotypes at 10, 18, and 26 years.

Mean (Min–Max) Standard Deviation	Children Not Wheezing at 10 Years (CNW10)	Children Wheezing at Age 10 Years (CW10)	*p*-Value
Prebronchodilator FEV1 (L)			
10 years	N = 480	N = 131	
	2.05 (1.21–3.03)	1.99 (1.25–3.00)	0.04 *
	0.30	0.31	
Prebronchodilator FVC (L)			
26 years	N = 347	N = 93	
	4.75 (2.83–8.08)	5.01 (3.53–7.10)	0.04 *
	1.23	1.21	
FEV1/FVC (%)			
10 years	N = 480	N = 131	
	0.89 (0.72–1.00)	0.87 (0.64–0.99)	<0.0002 * ‡
	0.05	0.07	
18 years	N = 463	N = 116	
	0.88 (0.58–1.00)	0.84 (0.57–1.00)	<0.0001 * ‡
	0.07	0.09	
26 years	N = 347	N = 93	
	0.81 (0.51–1.00)	0.78 (0.47–0.98)	<0.0005 *
	0.07	0.08	
FEF 25–75 (L/min)			
10 years	N = 480	N = 131	
	2.48 (0.95–3.96)	2.27 (0.84–3.52)	0.0002 *
	0.55	0.62	
18 years	N = 463	N= 116	
	4.51 (0.77–8.04)	4.21 (1.63–9.63)	0.01 *
	1.12	1.33	
FENO (mean)			
26 years	342	91	
	19.49 (5.00–254.00)	32.32 (5.00–228.00)	<0.002 *
	1.90	2.36	
BHR Dose–Response Slope (mean)			
10 years	N = 348	N = 131	
	1.35 (0.37–2.92)	1.80 (0.89–3.29)	<0.00001 *
	0.36	0.59	
18 years	N = 334	N = 92	
	1.06 (0.51–2.97)	1.26 (0.80–2.94)	<0.00001 *
	1.29	1.28	

Abbreviations: FEV1: forced expiratory volume in the first second, FVC: forced vital capacity, FEF 25–75: forced expiratory flow at 25–75%, FeNO: fractional exhaled nitric oxide, BHR: bronchial hyperresponsiveness. In this table, these values were studied: FEV1, FVC, FEV1/FVC, and FEF2575 at ages 10, 18, and 26 for the two groups CW10 and CNW10. Only statistically significant *p*-values are shown in the table. Log-transformation was made for not normally distributed variables. If normality was obtained, Student’s *t*-tests were performed on log-transformed data. ‡ Test made by Mann–Whitney U test when variables were not normally distributed after log-transformation. Student’s *t*-test was performed for each other characteristic that was normally distributed. * *p*-value for significance is 0.05.

**Table 3:** Evolution of lung function between 10 and 18 years and between 18 and 26 years for childhood wheezing phenotypes at 10 years.

	Difference 18–10 Years			Difference 26–18 Years		
	Children Not Wheezing at 10 Years	Children Wheezing at Age 10 Years	*p*-Value	Children Not Wheezing at 10 Years	Children Wheezing at Age 10 Years	*p*-Value
(*n* =)	403	106		305	81	
FEV1 Changing mean (sd)	1.95 (0.68)	1.94 (0.67)	0.89	0.03 (0.32)	−0.02 (0.34)	0.24
FVC Changing mean (sd)	2.25 (0.79)	2.37 (0.79)	0.16	0.39 (0.42)	0.41 (0.42)	0.57
FEV1/FVC Changing mean (sd)	−0.009 (0.06)	−0.02 (0.06)	0.09	0.08 (0.05)	0.06 (0.07)	0.08
(*n* =)	463	116		347	93	
FEF25–75 Changing mean (sd)	2.03 (0.70)	1.94 (0.45)	0.19	−0.6 (0.40)	−0.52 (0.34)	0.09
(*n* =)				342	91	
FENO Changing mean (sd)				−2.3 (1.05)	−12.8 (2.06)	<0.002 *
(*n* =)	334	92				
DRS Changing mean (sd)	−0.29 (0.19)	−0.54 (0.24)	<0.002 *			

Two-sample *t*-tests were performed for each characteristic. * *p*-value for significance is < 0.05.

**Table 4:** Comparison of early-life, 10-year, and 26-year characteristics of wheezing and nonwheezing phenotypes at 10 years that show significant association to wheezing outcome at 26 years.

Variable If Categorical No (%) If Numeric Mean (Min-Max)	Children Not Wheezing at Age 10 Years (*n* = 595) (55 Excluded)		*p*-Value	Children Wheezing at Age 10 Years (*n* = 146) (14 Excluded)		*p*-Value
	Never-Wheezers at 10, 18, and 26 Years (*n* = 444)	Wheezing at 26 Years (*n* = 96)		Wheezers at 10, 18, and 26 Years (*n* = 58)	Not Wheezing at 26 Years (*n* = 74)	
Early Childhood						
Recurrent chest infections at 2 years	7.6% (34/431)	14.1% (13/92)	0.004 *	20.0% (11/55)	55.6% (21/72)	0.24
Eczema at 4 years	17.6% (78/444)	31.3% (30/96)	0.02 *	37.9% (22/58)	29.7% (22/74)	0.32
Atopy at 4 years	13.1% (51/390)	13.6% (11/81)	0.90	54.6% (30/55)	34.4% (22/64)	0.03 *
Family history of rhinitis	46.3% (195/421)	59.8% (55/92)	0.02*	79.3% (46/58)	48.6% (35/72)	0.0003 *
Parents tobacco smoking at birth	32.7% (143/437)	44.7% (42/94)	0.03 *	36.2% (21/58)	40.8% (29/71)	0.59
10 years						
Dog at home at 10 years	38.9% (173/444)	27.1% (26/96)	0.03 *	44.8% (26/58)	41.9% (31/74)	0.74
Current asthma at 10 years	0.2% (1/444)	8.3% (8/96)	<0.00001 *	75.8% (44/58)	58.1%(43/74)	0.03 *
Current rhinitis at 10 years	10.7% (47/440)	11.5% (11/85)	0.54	56.9% (33/58)	27.1% (20/74)	0.0005 *
Atopic status at 10 years	18.6% (70/377)	16.7% (16/80)	0.48	72.4 (42/58)	50% (34/68)	0.01 *
Asthma treatment at 10 years	0.5% (2/396)	9.3% (8/86)	<0.00001 * †	62.2% (46/74)	84/2% (48/57)	0.006 * †
Inhaled corticosteroids (ICS) at 10 years	0.8% (3/396)	5.9% (5/85)	0.006 * †	1.8% (1/57)	7.9% (3/38)	0.03 * †
Bronchial hyperreactivity (BHR) at 10 year defined by PC20 <4 mg/ml (%)	12.7% (31/244)	9.1% (6/66)	0.52	61.4% (35/57)	35.9% (23/64)	0.005 *
26 years						
BMI at 26 years	26.1 (17.0–50.6)	25.8 (17.8–50.6)	0.65	26.8 (18.5–42.3)	24.4 (18.3–44.4)	0.025 *
Current asthma at 26 years	1.1% (5/443)	35.4% (34/96)	<0.00001 *	96.5% (55/57)	4.1%(3/74)	<0.00001 *
Current wheeze at 26 years	0% (0/444)	100% (96/96)	<0.00001 *	100% (58/58)	0% (0/74)	<0.00001 *
Current rhinitis at 26 years	33.1% (147/444)	61.5% (59/96)	<0.00001 *	72.4% (42/58)	48.7% (38/74)	0.014 *
Current eczema at 26 years	8.3% (37/444)	9.4% (9/96)	0.74	22.4% (13/58)	6.8% (5/73)	0.02 *
Atopic status at 26 years	37.2% (97/261)	49.1% (28/57)	0.09	84.8% (39/46)	29.7% (22/40)	0.002 *
Asthma treatment at 26 years	0.5% (2/443)	13.5% (13/96)	<0.00001 * †	36.2% (21/58)	2.7% (2/74)	<0.00001 * †
Inhaled corticosteroids (ICS) at 26 years	0.2% (1/443)	2.1% (2/96)	0.08†	15.5% (9/58)	0% (0/74)	0.0004 * †

A chi 2 test was performed for each characteristic. † Test made by Fisher’s exact test when necessary * *p*-value for significance is 0.05. Notes to Table 4: Participants included in the longitudinal outcome analysis of nonwheezers (CNW10) at 10 years (*n* = 595). Fifty-five participants were excluded who had transient wheezing at 18 years but no wheezing at 10 or 26 years. Participants included in the longitudinal outcome analysis of wheezers (CW10) at 10 years (*n* = 146). Fourteen participants were excluded who transiently did not wheeze at 18 years but wheezed at both 10 and 26 years. In this table, for the sake of consistency, we decided to present all the significant and nonsignificant *p*-value data between the different groups.

**Table 5:** Factors associated at multivariable regression with wheeze development or remission in adulthood among childhood wheeze phenotypes at 10 years.

Wheezing Phenotype	Associated Factor	OR	95% Confidence Interval	*p*-Value
A. Children not wheezing at age 10 years with wheezing development in adulthood	Eczema at age 4 years	2.19	(1.27–3.73)	<0.0005
	Family history of rhinitis	1.76	(1.09–2.90)	0.02
	Tobacco smoking from parents at birth	1.68	(1.03–2.74)	0.04
B. Childhood wheezing at age 10 years with wheezing remission in adulthood	Atopy at age 4 years	0.44	(0.19–0.96)	0.04
	Family history of rhinitis	0.23	(0.09–0.52)	0.0006

**Table 5A: With adult wheezing development.** Of the eligible cases, 82.7% (492/595) were included. Factors were entered into the models that had significance *p* < 0.2 at univariate analysis: recurrent chest infections at 2 years, eczema at 4 years, family history of rhinitis, parents’ tobacco smoking at birth, rhinitis at 4 years. **Table 5B: With wheezing remission in adulthood.** Of eligible cases, 90.4% (132/146) were included. Factors were entered the model that had significance *p* < 0.2 at univariate analysis: atopy at age 4 years, family history of rhinitis, low birthweight <2.5 kg, exposure to tobacco smoke at birth.

## Data Availability

After publication, the data will be made available to others on reasonable requests to the corresponding author. A proposal with detailed description of study objectives and statistical analysis plan will be needed for evaluation of the reasonability of requests. Additional materials might also be required during the process of evaluation. Deidentified participant data will be provided after approval from the corresponding authors.
